# Chromosome 9p21 in sporadic amyotrophic lateral sclerosis in the UK and seven other countries: a genome-wide association study

**DOI:** 10.1016/S1474-4422(10)70197-6

**Published:** 2010-10

**Authors:** Aleksey Shatunov, Kin Mok, Stephen Newhouse, Michael E Weale, Bradley Smith, Caroline Vance, Lauren Johnson, Jan H Veldink, Michael A van Es, Leonard H van den Berg, Wim Robberecht, Philip Van Damme, Orla Hardiman, Anne E Farmer, Cathryn M Lewis, Amy W Butler, Olubunmi Abel, Peter M Andersen, Isabella Fogh, Vincenzo Silani, Adriano Chiò, Bryan J Traynor, Judith Melki, Vincent Meininger, John E Landers, Peter McGuffin, Jonathan D Glass, Hardev Pall, P Nigel Leigh, John Hardy, Robert H Brown, John F Powell, Richard W Orrell, Karen E Morrison, Pamela J Shaw, Christopher E Shaw, Ammar Al-Chalabi

**Affiliations:** a King's College London, Medical Research Council Centre for Neurodegeneration Research, Department of Clinical Neuroscience, Institute of Psychiatry, London, UK; b Department of Medical and Molecular Genetics, Guy's Hospital, Institute of Psychiatry, London, UK; c Medical Research Council Centre for Social, Genetic and Developmental Psychiatry, Institute of Psychiatry, London, UK; d Department of Neuroscience, Institute of Psychiatry, London, UK; e University College London Institute of Neurology, Queen Square, London, UK; f Department of Neurology, Rudolf Magnus Institute of Neuroscience, University Medical Centre Utrecht, Netherlands; g Department of Neurology, University Hospital Leuven, and Laboratory for Neurobiology, Vesalius Research Center, VIB, University of Leuven, Leuven, Belgium; h Department of Neurology, Beaumont Hospital and Trinity College Dublin, Ireland; i Department of Clinical Neuroscience, Umeå University, Sweden; j Department of Neurology and Laboratory of Neuroscience, “Dino Ferrari” Centre, Università degli Studi di Milano—IRCCS Istituto Auxologico Italiano, Milano, Italy; k Department of Neuroscience, University of Turin, and Azienda Ospedaliera Universitaria San Giovanni Battista, Turin, Italy; l Neuromuscular Diseases Research Group, Laboratory of Neurogenetics, National Institute on Aging, National Institutes of Health, Bethesda, USA; m INSERM UMR-788 and University of Paris 11, Bicetre Hospital, Paris, France; n Assistance Publique—Hôpitaux de Paris, Université Pierre et Marie Curie, Hôpital de la Salpêtrière, Paris, France; o Department of Neurology, University of Massachusetts Medical School, Worcester, MA, USA; p Department of Neurology, Emory University, Atlanta, GA, USA; q School of Clinical and Experimental Medicine, College of Medicine and Dentistry, University of Birmingham, and Neurosciences Division, University Hospitals Birmingham NHS Foundation Trust, Birmingham, UK; r Academic Neurology Unit, Department of Neuroscience, Faculty of Medicine, Dentistry and Health, University of Sheffield, Sheffield, UK

## Abstract

**Background:**

Amyotrophic lateral sclerosis (ALS) is a neurodegenerative disease of motor neurons that results in progressive weakness and death from respiratory failure, commonly within about 3 years. Previous studies have shown association of a locus on chromosome 9p with ALS and linkage with ALS–frontotemporal dementia. We aimed to test whether this genomic region is also associated with ALS in an independent set of UK samples, and to identify risk factors associated with ALS in a further genome-wide association study that combined data from the independent analysis with those from other countries.

**Methods:**

We collected samples from patients with sporadic ALS from 20 UK hospitals and obtained UK control samples from the control groups of the Depression Case Control study, the Bipolar Affective Case Control Study, and the British 1958 birth cohort DNA collection. Genotyping of DNA in this independent analysis was done with Illumina HumanHap550 BeadChips. We then undertook a joint genome-wide analysis that combined data from the independent set with published data from the UK, USA, Netherlands, Ireland, Italy, France, Sweden, and Belgium. The threshold for significance was p=0·05 in the independent analysis, because we were interested in replicating a small number of previously reported associations, whereas the Bonferroni-corrected threshold for significance in the joint analysis was p=2·20×10^−7^

**Findings:**

After quality control, samples were available from 599 patients and 4144 control individuals in the independent set. In this analysis, two single nucleotide polymorphisms in a locus on chromosome 9p21.2 were associated with ALS: rs3849942 (p=2·22×10^−6^; odds ratio [OR] 1·39, 95% CI 1·21–1·59) and rs2814707 (p=3·32×10^−6^; 1·38, 1·20–1·58). In the joint analysis, which included samples from 4312 patients with ALS and 8425 control individuals, rs3849942 (p=4·64×10^−10^; OR 1·22, 95% CI 1·15–1·30) and rs2814707 (p=4·72×10^−10^; 1·22, 1·15–1·30) were associated with ALS.

**Interpretation:**

We have found strong evidence of a genetic association of two single nucleotide polymorphisms on chromosome 9 with sporadic ALS, in line with findings from previous independent GWAS of ALS and linkage studies of ALS–frontotemporal dementia. Our findings together with these earlier findings suggest that genetic variation at this locus on chromosome 9 causes sporadic ALS and familial ALS–frontotemporal dementia. Resequencing studies and then functional analysis should be done to identify the defective gene.

**Funding:**

ALS Therapy Alliance, the Angel Fund, the Medical Research Council, the Motor Neurone Disease Association of Great Britain and Northern Ireland, the Wellcome Trust, and the National Institute for Health Research Dementias and Neurodegenerative Diseases Research Network (DeNDRoN).

## Introduction

Amyotrophic lateral sclerosis (ALS) is a neurodegenerative disease of motor neurons that results in progressive weakness and death from respiratory failure, commonly within about 3 years. About one in 300 people^1^ develops ALS but its prevalence is low—five per 100 000 people—because of its poor prognosis. About 5% of patients have a family history of ALS, and occasionally frontotemporal dementia. ALS and frontotemporal dementia can coexist in the same family or individual.

Several studies have reported linkage of ALS–frontotemporal dementia with a locus on chromosome 9p, and specifically with a shared region that spans about 3·6 Mb.^2^, ^3^, ^4^, ^5^, ^6^, ^7^ A genome-wide association study (GWAS) reported that sporadic ALS was associated with single nucleotide polymorphisms (SNPs) in the same region of chromosome 9p.^8^ Therefore, this locus is the focus of research into the genetic basis of familial ALS, the relation between ALS and frontotemporal dementia, and the mechanisms underlying sporadic ALS and frontotemporal dementia.

Previous GWAS in ALS have reported association results that have been replicated within the same study but rarely in independent studies.^9^ For example, variation in the *ITPR2* gene was associated with ALS in a GWAS and was replicated within the study^10^ but has not been found by other investigators.^8^ Variation in *DPP6* was reported to be associated with ALS and was replicated within the same study,^11^ and a second study found that the same SNPs were the most strongly associated, although they did not achieve genome-wide significance.^12^ A subsequent study failed to confirm the findings.^13^ Variation in the *UNC13A* gene was strongly associated with ALS and was replicated within the same study,^8^ but no successful replication of the association with this locus has been reported.

We therefore aimed to examine previously reported genetic associations for ALS in a GWAS in an independent set of UK samples. We also aimed to combine these data with existing genotypic data from seven other countries to generate a large GWAS in ALS.

## Methods

### Samples

Adults attending one of 20 UK hospitals and who had been diagnosed with ALS by two consultant neurologists were invited to participate in the UK National DNA Bank for Motor Neuron Disease Research (MND DNA bank), a national collection of ALS DNA samples derived from whole blood. Collection began in 2004, and enrolment is ongoing. Patients had no family history of ALS at the time of sampling, were of self-reported white European ancestry, and had onset of weakness on or after January, 2002. Patients provided ethically approved written consent for blood to be drawn. Control samples for the independent analysis were obtained from two sources: the control groups of the Depression Case Control (DeCC) study^14^ and the Bipolar Affective Case Control Study (BACCS)^15^ and data deposited by Panos Deloukas from the Wellcome Trust Sanger Institute (Cambridge, UK) and published online from the British 1958 birth cohort DNA collection.

Case and control samples for the joint analysis were from the UK, USA, Netherlands, Belgium, France, Italy, Ireland, and Sweden.^8^, ^11^, ^16^, ^17^ Demographic, ascertainment, and quality control details of these samples have been published previously.^8^, ^11^, ^16^, ^17^ All participating individuals gave written informed consent, and patients were selected for sporadic ALS. Samples were matched for geographical origin and ethnic origin only.

This research was approved by the research ethics committees of each relevant institution for all cohorts.

### Procedures

DNA was extracted by use of standard methods at three centres within 1 week of the blood being drawn (usually on the same day) and was stored centrally at the UK DNA banking network in Manchester. We used a barcode-based sample tracking system to minimise the risk of clerical error.

Genotyping of DNA from samples held with the UK MND DNA bank was done with Illumina HumanHap550 BeadChips (Illumina, CA, USA) by UCL Genomics, London, UK. All DNA samples went through stringent quality control, and processing was done under full laboratory information management system control. The raw data were analysed with GenomeStudio (Illumina) and extracted for statistical analysis.

Quality control procedures were applied to individual and SNP data (webappendix pp 1–3). Individuals were excluded if more than 1% of their genotypic data was missing; if they had abnormal heterozygosity, poor cluster separation, or a sex assignment that conflicted with phenotypic data; if they shared more than 5% of alleles identical by descent with any other study member; or if they were of non-European ancestry. SNPs with minor allele frequency less than 1%, showing non-random missingness between genotypes or cases and controls, or showing departure from Hardy-Weinberg equilibrium (p<1×10^−6^) were excluded.

For the joint analysis, raw genotypes were obtained from public databases or directly from research groups and merged into a single file for analysis, with a covariate file listing the site of origin. Genotyping and quality control measures for the studies in the joint analysis have been published previously.^8^, ^11^, ^16^, ^17^

All data were deposited at the ALS online genetics database^18^ and the European genome-phenome archive.

### Statistical analysis

We did power analysis with the Genetic Power Calculator.^19^ For the independent analysis there was 80% power to detect a typical previously reported association with an odds ratio (OR) of 1·2 and minor allele frequency of 0·25 at p=0·05. We report power at p=0·05 in the independent analysis because, although we have done a GWAS, we are interested in replicating association at five specified loci (*FGGY, DPP6, ITPR2, UNC13A*, and chromosome 9p21.2). For the joint analysis, we investigated the possibility of new associations within the genome and therefore we corrected for multiple testing. We used Bonferroni correction, which assumes the tests of association at each of the 227 475 SNPs are independent; the corrected threshold is therefore 2·20×10^−7^ (0·05/227 475), and there was 83·5% power to detect a similar variant at the threshold of p=2·20×10^−7^. Our aim was to use all of the available samples.

For the joint analysis, population stratification was controlled for by principal component analysis in EIGENSTRAT.^20^ We used the Tracy-Widom distribution to calculate the number of significant principal components needed as covariates in subsequent analyses, using the twstats program of the Eigensoft package.^21^

We tested association of genotypes with ALS by logistic regression with 30 principal components as covariates in the program PLINK (version 1.07).^22^ We used the expectation-maximisation algorithm—as used in PLINK—for haplotype estimation. Haplotypic association with ALS was tested with a sliding window of varying sizes, including a window sufficient to include all SNPs in the haplotype block that contained the most strongly associated SNPs.

We imputed genotypes by use of Impute2,^23^ with data from the 1000 Genomes Project and the International HapMap as templates.^24^ Genotype imputation allows the prediction of genotypes at untyped SNPs by the correlation between SNP genotypes in different populations. Association analysis can then be done on the predicted genotypes. Association analysis was done with SNPTEST,^25^ which accounts for uncertainty in the imputed genotypes, using the first ten principal components as covariates (the maximum possible). The correction for population stratification in the imputed data will have been weakened by the use of fewer principal components; however, the use of Impute2 increased our ability to deal with genotype uncertainty. We did this to identify variants for follow-up genotyping in a focused region, and thus the trade-off was deemed worthwhile.

Population attributable risk was estimated as:

$$\frac{\left(p^{2}+2pqg_{AB}+q^{2}g_{BB}\right)-1}{p^{2}+2pqg_{AB}+q^{2}g_{BB}}$$

where *q* is the risk allele frequency, *p*=1–*q*, and genotype relative risk is denoted by *g*, with the subscript showing heterozygote or homozygote status. Penetrance for each genotype was estimated as:

$$K\frac{f_{case}}{f_{control}}$$

where *K* is the lifetime prevalence and *f* is the genotype frequency.

### Role of the funding source

The sponsor of the study had no role in study design, data analysis, data interpretation, writing of the report, or the decision to submit the paper for publication. The Motor Neurone Disease Association is the sponsor of the UK National MND DNA bank, and therefore had a role in data collection. The corresponding author had full access to all of the data in the study and had final responsibility for the decision to submit the paper for publication.

## Results

For the independent analysis, genotypes were obtained from 663 individuals in the UK MND DNA bank, 1589 control samples from the DeCC study and BACCS, and 2930 control samples from the 1958 birth cohort. After quality control measures, samples were available from 599 people with ALS and 4144 control individuals. Table 1 summarises clinical features of the ALS cohort in the independent analysis.

**Table 1:** Clinical features of patients with amyotrophic lateral sclerosis in the independent analysis

		**Case cohort (n=599)**
Presentation*		
	Bulbar symptoms	165 (28%)
	Limb weakness	375 (63%)
	Respiratory	11 (2%)
	Mixed†	42 (7%)
Age of onset‡ (years)		60·7 (61·7, 22·9–86·5)
Diagnostic delay§ (months)		21·5 (18·3, 2·0–80·1)

Data are number (%) or mean (median, range).

* Data missing for six patients.

† Simultaneously reported weakness of limbs and bulbar musculature.

‡ Data missing for three patients.

§ Time between symptom onset and diagnosis. Data missing for eight patients.

For the joint analysis, genotypes were obtained from 4312 patients with ALS and 8425 control individuals. After quality control measures, samples were available from 4133 patients with ALS and 8130 control individuals, which were typed for 227 475 SNPs to generate 2 789 513 662 genotypes. Table 2 and the webappendix p 4 summarise demographic data.

**Table 2:** Characteristics of the component populations for the joint analysis after quality control

	**Cases**	**Controls**	**Total**	**Men**
Belgium^8^	286	307	593	313
France^16^	230	700	930	805
Netherlands^8^, ^10^, ^12^	977	988	1965	1152
Ireland^11^	210	200	410	219
Italy^17^	259	241	500	271
Sweden^8^	422	441	863	481
USA^16^	923	904	1827	1061
UK^16^	227	205	432	264
UK MND DNA bank	599	0	599	382
DeCC study and BACCS	0	1505	1505	567
1958 birth cohort	0	2639	2639	1357
Total	4133	8130	12 263	6872

The final three rows refer to samples used in the independent study. DeCC=Depression Case Control. BACCS=Bipolar Affective Case Control Study.

**Figure 1:**
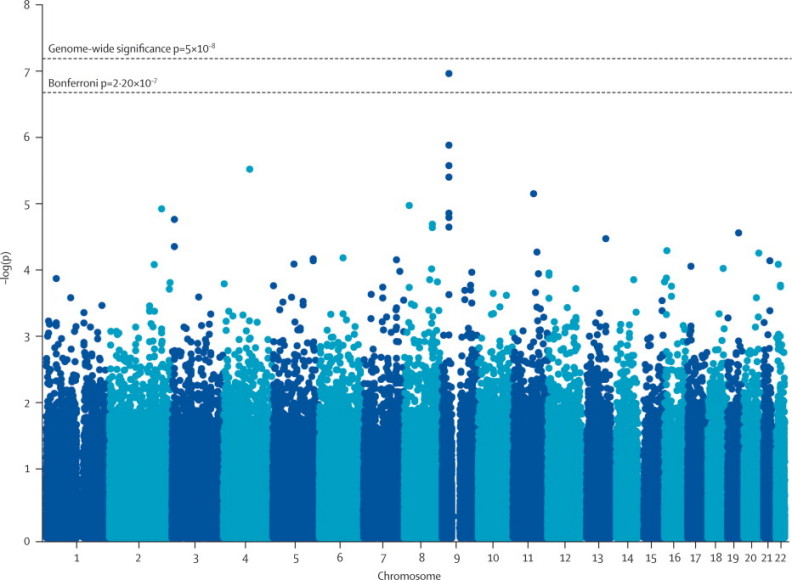
p values for association in the independent genome-wide association study

In the independent analysis, SNPs rs3849942 (p=2·22×10^−6^; OR 1·39, 95% CI 1·21–1·59) and rs2814707 (p=3·32×10^−6^; 1·38, 1·20–1·58) on chromosome 9 showed association with ALS (figure 1, table 3, webappendix pp 5–6). rs903603, which had strong linkage disequilibrium with the associated SNPs (*D*'=0·98), was the SNP most strongly associated with ALS in the independent set (p=8·92×10^−8^; OR 0·71, 95% CI 0·63–0·81). No other loci achieved genome-wide significance.

**Table 3:** Associations for genes reported to be associated with amyotrophic lateral sclerosis in previous genome-wide association studies

	n for associated SNP in previous studies*	**Associated SNP**	**Controls**	**p value in previous studies**	**Independent analysis**†			**Joint analysis**‡		
					**p value**	**Minor allele frequency**		**p value**	**Minor allele frequency**	
						Cases	Controls		Cases	Controls
*FGGY*^9^	1287	rs6700125	1567	6·0×10^−4^	0·71	0·342	0·337	0·08	0·351	0·339
*DPP6*^12^	1767	rs10260404	1916	5·4×10^−8^	0·89	0·397	0·395	0·08	0·391	0·380
*ITPR2*^10^	1337	rs2306677	1356	3·28×10^−6^	0·98	0·100	0·100	0·97	0·100	0·096
*UNC13A*^8^	4855	rs12608932	14 953	2·53×10^−14^	0·42	0·366	0·354	5·14×10^−4^	0·382	0·344
9p21.2^8^	4855	rs2814707	14 953	7·45×10^−9^	3·32×10^−6^	0·305	0·243	4·72×10^−10^	0·272	0·236
9p21.2^8^	4855	rs3849942	14 953	1·01×10^−8^	2·22×10^−6^	0·304	0·241	4·64×10^−10^	0·271	0·235
9p21.2^8^	4855	rs903603	14 953	..	8·92×10^−8^	0·411	0·493	1·37×10^−7^	0·456	0·492

Previously reported significant associations and the results we obtained for those genes in the two parts of this study are shown.^8^, ^9^, ^10^, ^12^

* Total combined sample for the reported SNP, which includes a GWAS component and a non-GWAS follow-up study.

† 599 cases and 4144 controls.

‡ 4133 cases and 8130 controls.

**Figure 2:**
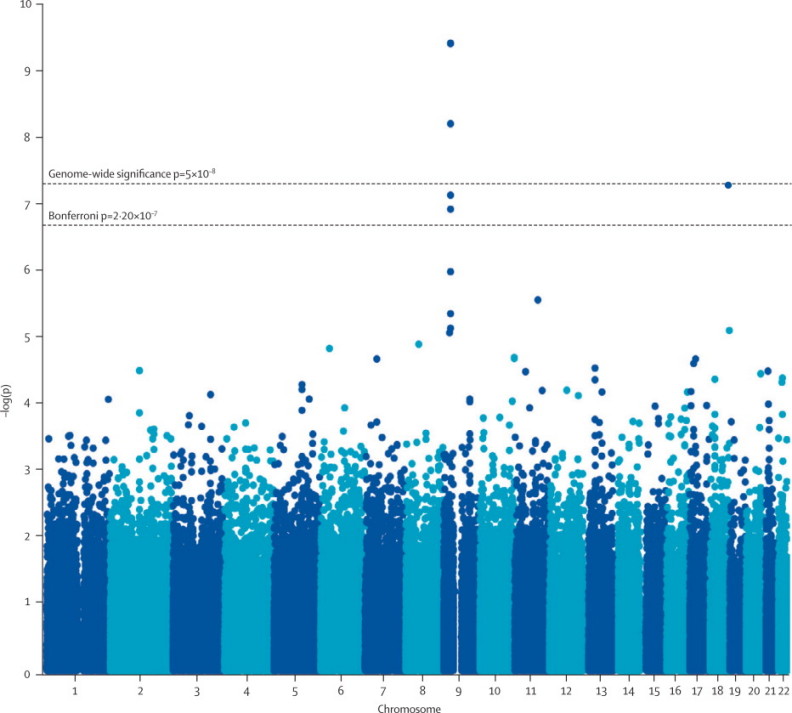
p values for association in the joint analysis

In the joint analysis, rs3849942 (p=4·64×10^−10^; OR 1·22, 95% CI 1·15–1·30), rs2814707 (p=4·72×10^−10^; 1·22, 1·15–1·30), and rs903603 (p=1·37×10^−7^; 0·86, 0·82–0·91) showed association with ALS (figure 2, table 3, webappendix pp 7–8). SNPs at other loci that were associated with ALS in previous studies did not reach significance in the joint analysis, except the previously associated SNP in *UNC13A* (rs12608932; table 3). The genotype relative risk for rs3849942 was 1·24 (heterozygote) and 1·49 (homozygote), with minor allele frequencies of 0·27 in patients with ALS and 0·24 in controls. The population attributable risk of rs3849942 was 0·10 and the penetrance of the risk allele was 0·004 (heterozygote) and 0·005 (homozygote).

A haplotypic association of rs3849942 and rs10122902 (omnibus p=1·51×10^−11^) was identified in the joint analysis. Of the three haplotypes in the population (GA, AG, and GG), one had equal frequency in people with ALS and controls (GA: frequency cases 0·21, controls 0·20; p=0·123), one was associated with increased risk of ALS (AG: frequency cases 0·27, controls 0·23, p=7·76×10^−10^), and was one associated with reduced risk of ALS (GG: frequency cases 0·52, controls 0·57; p=4·83×10^−11^). Similar results were obtained for the independent study: rs3849942 and rs10122902 showed haplotypic association (omnibus p=1·01×10^−7^), and the same three haplotypes had similar control frequencies (GA: frequency cases 0·21, controls 0·19, p=0·074; AG: frequency cases 0·30, controls 0·24, p=3·18×10^−6^; GG: frequency cases 0·48, controls 0·57, p=4·24×10^−8^). Longer SNP haplotypes did not result in stronger association in either part of the study (data not shown). The sliding window showed that pairs of SNPs within a 106·5 kb block of linkage disequilibrium flanked by rs4879515 and rs702231 were nearly all associated with ALS at p<1×10^−3^, whereas outside this region there were no significant associations.

**Figure 3:**
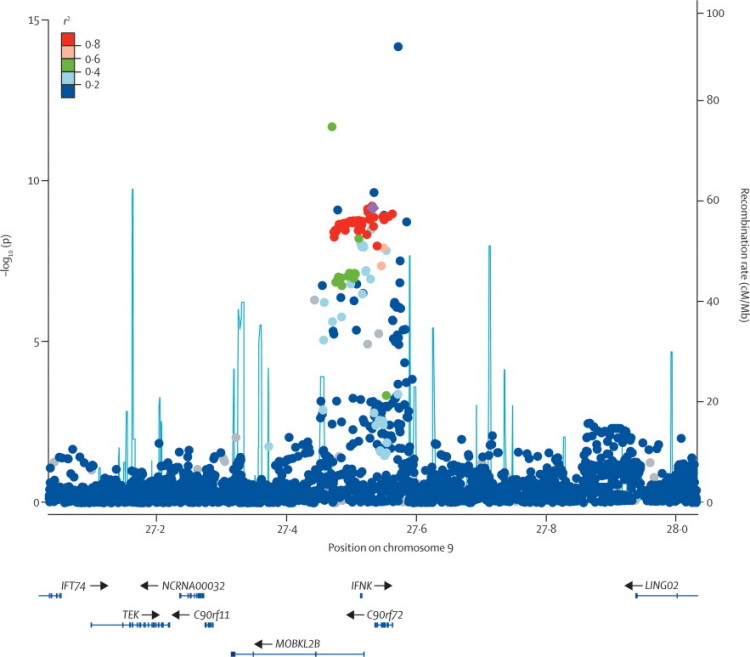
Genetic architecture of the associated region Circles=−log_10_ of the p value of association with amyotrophic lateral sclerosis for typed and imputed single nucleotide polymorphisms in the joint analysis, coloured according to linkage disequilibrium with rs3849942 (shown as a purple diamond) as measured by *r*^2^. Blue lines=recombination rate across the locus based on the 1000 genomes project. Genes in the region are displayed below the graph. Arrows indicate the direction of transcription.

Imputation in the joint analysis in the region of interest (ie, the 3·6 Mb region identified by linkage) gave results for 21 899 imputed SNPs that were derived from 519 typed SNPs. Five SNPs were more strongly associated with ALS than was rs3849942; the most strongly associated SNP was a rare variant, chr9:27573575 (minor allele frequency 0·054; p=6·40×10^−15^). The most strongly associated variant with a minor allele frequency greater than 0·1 was rs13295103 (p=1·97×10^−12^; figure 3).

## Discussion

In our independent study we have confirmed association of a chromosome 9 locus with ALS. We did not replicate any of the other previously reported associations, suggesting that our study gave a false-negative result or that they were population specific or false-positive results (panel, table 3).^8^, ^9^, ^10^, ^11^, ^12^ In our joint analysis, which is the largest GWAS of ALS to date, chromosome 9p21.2 and *UNC13A* were the only significantly associated loci. The association of *UNC13A* with ALS was weaker in the joint analysis than in the study that identified the association,^8^ which was well powered and replicated the results within the same study. Our independent samples do not show the association, possibly because the *UNC13A* variation is not a major cause of ALS in the UK, and so adding in these samples to the joint analysis we inevitably weaken the previously reported association. Given that the heritability of sporadic ALS is 0·61 (95% CI 0·38–0·78),^26^ our findings suggest that even larger GWAS are required to have sufficient power to detect genetic variations responsible for ALS. A potential limitation of this study is that not all control individuals were age and sex matched, although they were matched for geographical origin and ancestry. For a disease as rare as ALS, age and sex matching does not increase statistical power, whereas it would for a more common disorder.

Panel **Research in context**

**Systematic review**

To identify previously published genome-wide association studies (GWAS) in amyotrophic lateral sclerosis (ALS), we searched PubMed and the ALSOD database (http://www.alsod.iop.kcl.ac.uk), and contacted known ALS genetics research groups. Four GWAS reported findings that reached genome-wide significance;^8^, ^9^, ^10^, ^11^, ^12^ we designed the independent study to replicate these. Association studies were selected for the joint analysis if research diagnostic criteria for ALS were used, if microarrays of at least 300 000 single nucleotide polymorphisms were used, and if individual rather than pooled genotypes were available. Because the main finding of the independent and joint analyses was of an association to a region on chromosome 9 also known to be associated with familial ALS–frontotemporal dementia and sporadic frontotemporal dementia, we searched PubMed and contacted research groups and identified a further seven families with ALS–frontotemporal dementia^2^, ^3^, ^4^, ^7^ and a GWAS of frontotemporal dementia.^29^ These linkage data have been well summarised in a linkage paper.^4^

**Interpretation**

The ALS-associated locus on chromosome 9p was independently replicated, and no other loci were identified, suggesting that even larger genome-wide association studies are needed. We have shown the association signal on chromosome 9p lies in a well circumscribed block of linkage disequilibrium of about 106·5 Kb in size, which is flanked by markers rs4879515 and rs702231 and includes three annotated genes. This locus has also previously been implicated in sporadic frontotemporal dementia and in families with ALS–frontotemporal dementia. Because there is pathological and clinical overlap of ALS and frontotemporal dementia, the most logical interpretation is that dysfunction of a single gene product is responsible for some cases of ALS, frontotemporal dementia, and ALS–frontotemporal dementia.

The associated 9p21 locus is the focus of much interest for researchers of both ALS and frontotemporal dementia. Although frontotemporal dementia and ALS are independent diseases, there is some overlap. About 5% of people with ALS have overt frontotemporal dementia and up to 51% have subtle cognitive deficits that suggest frontal and temporal lobe dysfunction.^27^ Conversely, about 50% of people with frontotemporal dementia have features of motor neuron degeneration.^28^ A locus on chromosome 9 has been independently linked to ALS–frontotemporal dementia in seven families;^2^, ^3^, ^4^, ^5^, ^6^, ^7^ this 3·6 Mb locus is defined across studies by the flanking markers D9S169 and D9S251. The SNPs we have identified lie within this region, with the peak association at 106·5 Kb. A GWAS that used pathological subtyping of patients with frontotemporal dementia to increase homogeneity also identified the same SNPs as those most strongly associated with sporadic frontotemporal dementia (rs3849942 p=1·38×10^−6^ and rs2814707 p=5·72×10^−6^).^29^ Therefore, variation in the same gene is likely to be responsible for sporadic ALS, sporadic frontotemporal dementia, and familial ALS–frontotemporal dementia. Assuming that the association locus is the same as the linkage locus, the region of interest in families with ALS–frontotemporal dementia must now be thought to be much narrower than that defined by recombination; however, if a synthetic association of rare variants and ALS is being detected, the disease-causing variation could still lie a substantial distance from the association peak.^30^

Penetrance is about 20% in the family with the strongest association between chromosome 9 and ALS–frontotemporal dementia.^3^ Thus, familial variants might underlie at least some of the sporadic cases of ALS and frontotemporal dementia. The large signal seen in our independent study of UK samples is consistent with a founder effect in the UK population, which possibly extends out to other populations. In this respect, that a GWAS of a cohort of Finnish patients with ALS and controls also detected the chromosome 9p locus (rs3849942 p=9·11×10^−11^) is interesting.^31^ This association signal was driven mainly by people with familial ALS; the most strongly associated SNP in this subset of patients was rs2225389 (p=2·23×10^−12^). All the patients with familial ALS who were not homozygous for the D90A allele of the *SOD1* gene, and almost 20% of the patients with sporadic ALS, shared a 42-SNP haplotype, which strongly suggests a founder effect for the chromosome 9p locus, at least in Finland. However, the penetrance of the associated risk allele of rs3849942 in sporadic ALS in our study is only slightly higher than the background risk of ALS (0·36–0·45%),^1^ whereas the penetrance of the familial variants is about 20%; thus, the likelihood that the same variation is responsible for both familial and sporadic ALS is questionable, which makes a founder effect less likely. The significant association of rs3849942 with sporadic ALS in our joint analysis including multiple populations suggests that a common genetic variation might be contributing to disease risk. The two-SNP haplotype that was most strongly associated with ALS in the joint analysis (rs3849942-rs10122902) mimics the behaviour of the *APOE* association with Alzheimer's disease; the three *APOE* alleles—ɛ2, ɛ3, and ɛ4—are in fact pairs of alleles at two SNPs forming a haplotype. When a haplotype is formed from alleles at two SNPs there are four possible haplotypes, although not all will necessarily exist in the population. For both the rs3849942-rs10122902 haplotype in ALS and the *APOE* haplotype in Alzheimer's disease, only three of the four possible haplotypes are present, and the fourth does not exist in the population. In both cases, all identified haplotypes are common, and one is associated with risk of the disease (*APOE* ɛ4 in Alzheimer's disease; AG in ALS), one is associated with protection from the disease (*APOE* ɛ2 in Alzheimer's disease; GG in ALS), and one is neutral (*APOE* ɛ3 in Alzheimer's disease; GA in ALS). Thus, common variation, as with *APOE* in Alzheimer's disease, rather than a founder effect, could be responsible for sporadic ALS. However, because the penetrance at a functional variant might be much higher than at an associated tag SNP, which would account for the discrepancy between the penetrance for familial ALS and sporadic ALS, a founder effect cannot be excluded. There must be multiple variants worldwide because families with chromosome 9p-linked ALS–frontotemporal dementia do not all share a common haplotype, and so multiple founders must exist. However, the chromosome 9p locus is important in ALS because the population attributable risk estimate shows that it is involved in 10% of all cases of sporadic ALS.

The disease-associated SNPs we have identified lie between two genes, *C9orf72* and *MOBKL2B*, and are close to *IFNK*, which lies in an intron of *MOBKL2B*. The protein encoded by *MOBKL2B* is Mps one binder kinase activator-like 2B, which is involved in kinase regulation. On the opposite strand at the same location is *IFNK*, which encodes interferon κ precursor, which is important for immunity to viral infection. The SNPs comprising the most strongly associated haplotype (rs3849942 and rs10122902) and imputed SNP (chr9:27573575) overlap or lie within *C9orf72*, which encodes a protein that might be involved in cell development and possibly spermatogenesis.^32^

Although linkage of ALS–frontotemporal dementia to this region of chromosome 9 was first identified in 2006,^2^, ^3^ sequencing has not yet yielded a disease-causing variant such as a non-synonymous SNP or splice-site variation. If this remains the case, one explanation could be that the variation is tagged by SNPs but not easily detected by exome capture and high throughput sequencing or Sanger sequencing. The disease-causing variant might be in an unknown exon or gene and has therefore not been sequenced, or it might be a trinucleotide repeat expansion in an intron, although such repeats are not seen in the surrounding region. Copy number variation is not necessarily easily detected, depending on the sequencing method used, but three microarray-based studies in ALS have not detected ALS-associated copy number variation in this region of chromosome 9.^33^, ^34^, ^35^ Finally, a microRNA or non-annotated gene that has not been examined, or an inversion, which would be difficult to detect by sequencing, might be the disease-causing variant. Our results suggest genetic variation at this locus on chromosome 9 causes sporadic amyothophic lateral sclerosis and familial ALS–frontotemporal dementia. Resequencing studies and then functional analyses are needed to identify the defective gene.

## Web Extra Material

Supplementary webappendix
