# Supplementary material for: Chromosome 9p21 in sporadic amyotrophic lateral sclerosis in the UK and seven other countries: a genome-wide association study
Source: Lancet Neurol. 2010 Oct;9(10):986–94. doi: 10.1016/S1474-4422(10)70197-6 (PMC3257853; doi:10.1016/S1474-4422(10)70197-6)
Supplement: Supplementary webappendix [file mmc1.pdf]

THE LANCET Neurology

Supplementary webappendix

This webappendix formed part of the original submission and has been peer reviewed. We post it as supplied by the authors.

This online publication has been corrected. The corrected version first appeared at TheLancet.com/neurology on February 21, 2011.

Supplement to: Shatunov A, Mok K, Newhouse S, et al. Chromosome 9p21 in sporadic amyotrophic lateral sclerosis in the UK and seven other countries: a genome-wide association study. *Lancet Neurol* 2010; published online Aug 31. DOI:10.1016/S1474-4422(10)70197-6.

Webappendix
Web extra material for: Shatunov A, et al. Chromosome 9p21.2 is a replicable susceptibility locus for sporadic ALS

Detailed quality control protocol
Quality control filters were applied to all samples simultaneously.

1. Merging datasets
SNP positions were updated to genome build NCBI36.3 (equivalent to hg18) and SNP strand aligned for all datasets; a common map was generated and datasets merged.*
14143 individuals
4910 cases, 9233 controls
7937 males, 6204 females
265468 markers in common*

2. Initial sample quality control
Sample sex mismatch
The reported sex and the genetic sex were tested for mismatch.

59 individuals removed.
14084 individuals remaining
4872 cases, 9212 controls
7909 males, 6175 females

Exclusion of control individuals listed for exclusion by the Wellcome Trust Case Control Consortium (WTCCC)
225 individuals removed
13859 individuals remaining
4872 cases, 8987 controls
7795 males, 6064 females

Exclusion of case individuals with non-white European ancestry
To reduce the effects of population stratification, only those of white European ancestry were included in the analysis.

15 individuals removed
13844 individuals remaining
4857 cases, 8987 controls
7786 males, 6058 females

3. Initial marker quality control
Exclusion of markers listed for exclusion by WTCCC
350 markers removed
265118 markers remaining.

Exclusion of markers showing non-random missing status between cases and controls
Threshold for exclusion, $P = 1 \times 10^{-5}$.

11419 markers removed
253692 SNPs remaining.

Exclusion of SNPs showing non-random missing status by genotype
This test was performed by analysing haplotypes around each SNP. Threshold for exclusion, $P = 1 \times 10^{-5}$.

20477 markers removed
233222 markers remaining

Exclusion of SNPs with low minor allele frequency, low call rates and not in Hardy-Weinberg equilibrium in controls.
Thresholds for exclusion: minor allele frequency < 0·001, call rate < 95%, Hardy Weinberg test, $P = 1 \times 10^{-6}$.

0 markers removed for low frequency
12 markers removed for low call rate
179 markers removed for failing Hardy-Weinberg test in controls.
233031 markers remaining

4. Further general quality control measures
Exclusion of individuals with low genotyping rates
Threshold for exclusion, genotyping rate < 95%.
13 individuals removed
13831 individuals remaining
4847 cases, 8984 controls
7783 males, 6048 females
Total genotyping rate in remaining individuals 0.998633

Assessment of SNPs with outlier* P*-values
Threshold for assessment, $P \ll 1 \times 10^{-100}$.

15 markers removed
233016 SNPs remaining

Exclusion of chromosome X markers
5541 markers removed
Final count:
227475 markers remaining

5. Tests using a reduced set of SNPs to identify relatedness
These tests use a set of SNPs pruned to be in approximate linkage equilibrium (in our set, 74011 markers).

Exclusion of individuals with low heterozygosity
If the observed heterozygosity is lower than expected, this suggests inbreeding or poor genotyping quality. Threshold for exclusion, $P < 0{\cdot}05$.

84 individuals removed
12791 individuals left

Exclusion of outliers for ancestry
A multidimensional scaling plot was derived from the reduced set of SNPs. The first two axes were used to plot individuals based on ancestry. Those outside the main cluster were excluded.

54 individuals removed
12737 individuals left

Exclusion of cryptically related individuals
Using the statistical genetics measure of relatedness, pi-hat, which is the proportion of genetic variants identical by descent between two individuals. Thus, identical twins or duplicate samples, show pi-hat = 1, siblings show pi-hat = 0·5 etc. Threshold for exclusion, pi-hat > 0·05. One individual of each pair was removed randomly.

474 individuals removed.

Final counts:
12263 individuals remaining
4133 cases, 8130 controls
6873 males, 5390 females

*SNP rs12608932 was missing from the DeCC and BACC control set (listed as UK_MDEPR in Tables 2 and S1), but passed all other quality control measures. It was therefore retained to allow study of the *UNC13A* association. The loss of data for 1505 controls reduced power for detection of this SNP, with the equivalent values to those given in the Results as 0·73 power in the independent data and 0·77 power in the joint analysis.

6. Controlling for population substructure

The Eigenstrat package was used to analyse population substructure, using genotypes at 74011 SNPs as covariates to generate principal components axes (PCAs) for all 12791 individuals. The Twstats package was used to determine the optimal number of PCAs to use. This uses the Tracy-Widom distribution to estimate the optimal number of PCAs to include. Correction for substructure was not necessary in the independent analysis ($\lambda_{GC} = 1 \cdot 01$). In the joint analysis, correction was essential because of the multiple populations studied. After correction, $\lambda_{GC} = 1 \cdot 04$, which suggests no inflation of the test statistic. The QQ plots provide additional evidence for this (Figures S1 and S2).

Country	*N*	Cases	Controls	Male	Female	SNPs genotyped
**Belgium**	645	304	341	330	315	317503
**France**	975	251	724	836	139	307790
**Netherlands**	2113	1046	1067	1250	863	535468
**Ireland**	432	221	211	231	201	561466
**Italy**	540	277	263	291	249	535468
**Sweden**	959	475	484	538	421	317503
**USA**	1920	967	953	1110	810	307790
**UK**	466	245	221	285	181	307790
**UK_MND**	663	663	0	424	239	584414
**UK_MDEPR**	1589	0	1589	596	993	473508
**UK_WTCCC**	2930	0	2930	1509	1421	1155595
**Total:**	**13232**	**4449**	**8783**	**7400**	**5832**	

Supplementary Table S1. Characteristics of the component populations for the joint analysis before quality control.
The last three rows refer to the independent study samples. UK refers to previously reported UK samples. For the independent study, UK_MND refers to the UK National MND DNA Bank samples, UK_MDEPR refers to controls from the DeCC and BACC studies and UK_WTCCC refers to control samples from the 1958 Birth Cohort.

Chromosome	SNP	bp	Minor allele	Odds ratio	*P*
9	rs903603	27519316	T	0·7132	8·92 x10^{-8}
9	rs774359	27551049	C	1·391	1·09 x 10^{-6}
9	rs3849942	27533281	A	1·387	2·22 x 10^{-6}
4	rs2866197	101325339	C	0·6776	2·52 x 10^{-6}
9	rs2814707	27526397	A	1·378	3·32 x 10^{-6}
11	rs1522659	80518746	A	0·727	5·93 x 10^{-6}
8	rs7003470	19386565	A	1·455	8·91 x 10^{-6}
2	rs2177083	202669910	C	0·7612	1·01 x 10^{-5}
9	rs7046653	27480967	A	1·334	1·17 x 10^{-5}
9	rs1982915	27569560	A	0·7623	1·37 x 10^{-5}
3	rs4684627	9231398	C	0·731	1·45 x 10^{-5}
8	rs1494913	108957826	G	0·7525	1·73 x 10^{-5}
9	rs10757665	27547919	C	0·7128	1·90 x 10^{-5}
8	rs1389976	108959180	G	0·7539	1·94 x 10^{-5}
19	rs11669124	44315092	A	0·7518	2·34 x 10^{-5}
13	rs878765	93387165	C	1·316	2·84 x 10^{-5}
3	rs11131163	9266861	G	0·7504	3·76 x 10^{-5}
16	rs8053509	8769772	G	1·41	4·33 x 10^{-5}
11	rs606087	93162290	A	0·6233	4·60 x 10^{-5}
20	rs911168	58430478	C	1·314	4·70 x 10^{-5}
6	rs9342307	92699953	T	0·7704	5·59 x 10^{-5}
5	rs1614336	157484446	C	1·286	5·80 x 10^{-5}
7	rs3736626	129137406	G	1·284	5·97 x 10^{-5}
21	rs2836770	39236574	G	1·277	6·19 x 10^{-5}
5	rs1173472	157488401	T	1·367	6·19 x 10^{-5}
5	rs1423515	82098300	A	1·597	6·99 x 10^{-5}
22	rs6004919	24785216	T	1·495	7·01 x 10^{-5}
2	rs3769185	173721745	T	0·7769	7·08 x 10^{-5}
17	rs17680211	14112411	A	1·69	7·51 x 10^{-5}
18	rs1517166	59848277	G	0·7842	8·11 x 10^{-5}
8	rs2957422	106345540	G	0·6978	8·24 x 10^{-5}
7	rs1527307	141357368	T	1·282	9·00 x 10^{-5}
9	rs1359637	115575231	T	0·7838	9·25 x 10^{-5}
12	rs7298545	4542187	C	1·295	9·51 x 10^{-5}
11	rs2726355	98960271	C	0·7092	9·75 x 10^{-5}
11	rs7117574	98967675	A	0·6517	9·79 x 10^{-5}

Supplementary Table S2. SNP associations at *P* < 1 x 10^{-4} in the independent study based on an additive model in logistic regression.
Five of the top ten SNPs are in the associated chromosome 9 region.

Chromosome	SNP	bp	Minor allele	Odds ratio	*P*
9	rs3849942	27533281	A	1·219	4·64 x 10^{-10}
9	rs2814707	27526397	A	1·218	4·72 x 10^{-10}
9	rs774359	27551049	C	1·196	7·30 x 10^{-9}
9	rs7046653	27480967	A	1·175	8·48 x 10^{-8}
19	rs12608932	17613689	C	1.174	5·14 x 10^{-8}
9	rs903603	27519316	T	0·8627	1·37 x 10^{-7}
9	rs4879515	27472235	T	1·144	1·16 x 10^{-6}
11	rs1488902	88984673	C	1·145	3·06 x 10^{-6}
9	rs1982915	27569560	A	0·8811	4·89 x 10^{-6}
9	rs10511816	27458461	T	1·134	8·05 x 10^{-6}
18	rs4799088	75674536	G	0·8694	8·69 x 10^{-6}
9	rs7852159	23565805	G	0·8813	9·40 x 10^{-6}
8	rs6999204	49730050	C	1·289	1·39 x 10^{-5}
6	rs6900980	34079204	T	1·168	1·62 x 10^{-5}
10	rs7904594	133584661	T	1·143	2·19 x 10^{-5}
10	rs10870270	133604324	A	1·142	2·25 x 10^{-5}
7	rs788718	45973069	T	0·859	2·29 x 10^{-5}
17	rs9909055	23620255	T	0·8706	2·30 x 10^{-5}
17	rs9944524	16153375	G	0·8901	2·68 x 10^{-5}
13	rs2256363	43716006	T	1·143	3·17 x 10^{-5}
2	rs3923566	113422642	C	0·868	3·43 x 10^{-5}
21	rs1735970	22045960	A	0·8785	3·50 x 10^{-5}
11	rs11823636	41488602	C	0·8647	3·57 x 10^{-5}
20	rs13045889	55858625	T	1·197	3·82 x 10^{-5}
22	rs470072	30593131	T	1·12	4·43 x 10^{-5}
18	rs1652373	19482645	C	1·122	4·64 x 10^{-5}
13	rs9533799	43706174	A	1·123	4·69 x 10^{-5}
22	rs136287	29522023	A	1·145	5·11 x 10^{-5}
5	rs2161123	109169426	G	1·163	5·56 x 10^{-5}
5	rs3797680	109188150	C	1·161	6·54 x 10^{-5}
12	rs2468411	67260873	G	1·125	6·72 x 10^{-5}
11	rs2017912	106578630	T	1·316	6·83 x 10^{-5}
17	rs5862	5343681	G	0·895	7·07 x 10^{-5}
16	rs9923415	79492360	G	1·31	7·19 x 10^{-5}
13	rs9542376	70026751	A	1·14	7·21 x 10^{-5}
3	rs2569944	145627635	C	1·116	7·83 x 10^{-5}
12	rs11613438	111964893	A	0·89	8·13 x 10^{-5}
5	rs9327807	137378377	C	0·8639	9·07 x 10^{-5}
9	rs10760706	101763513	C	1·123	9·14 x 10^{-5}
1	rs1776148	240109168	A	1·119	9·20 x 10^{-5}
10	rs4980270	124941369	T	1·172	9·77 x 10^{-5}
9	rs1997368	101753401	C	1·122	9·94 x 10^{-5}

Supplementary Table S3. SNP associations at *P* < 1 x 10^{-4} in the joint analysis based on an additive model in logistic regression with 30 principal component axes to correct for population substructure.

Eight of the top ten SNPs are in the associated chromosome 9 region.

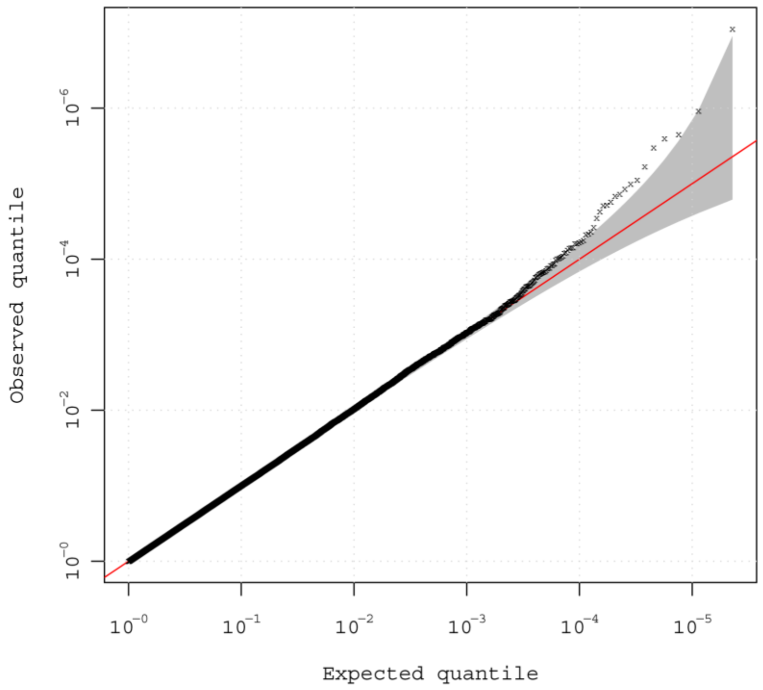

Supplementary Figure S1. Q-Q plot for the independent study.
The grey shaded area shows the 95% confidence limits for the expectation under the null hypothesis, shown in red. The test statistic is not inflated: $\lambda_{GC} = 1 \cdot 01$.

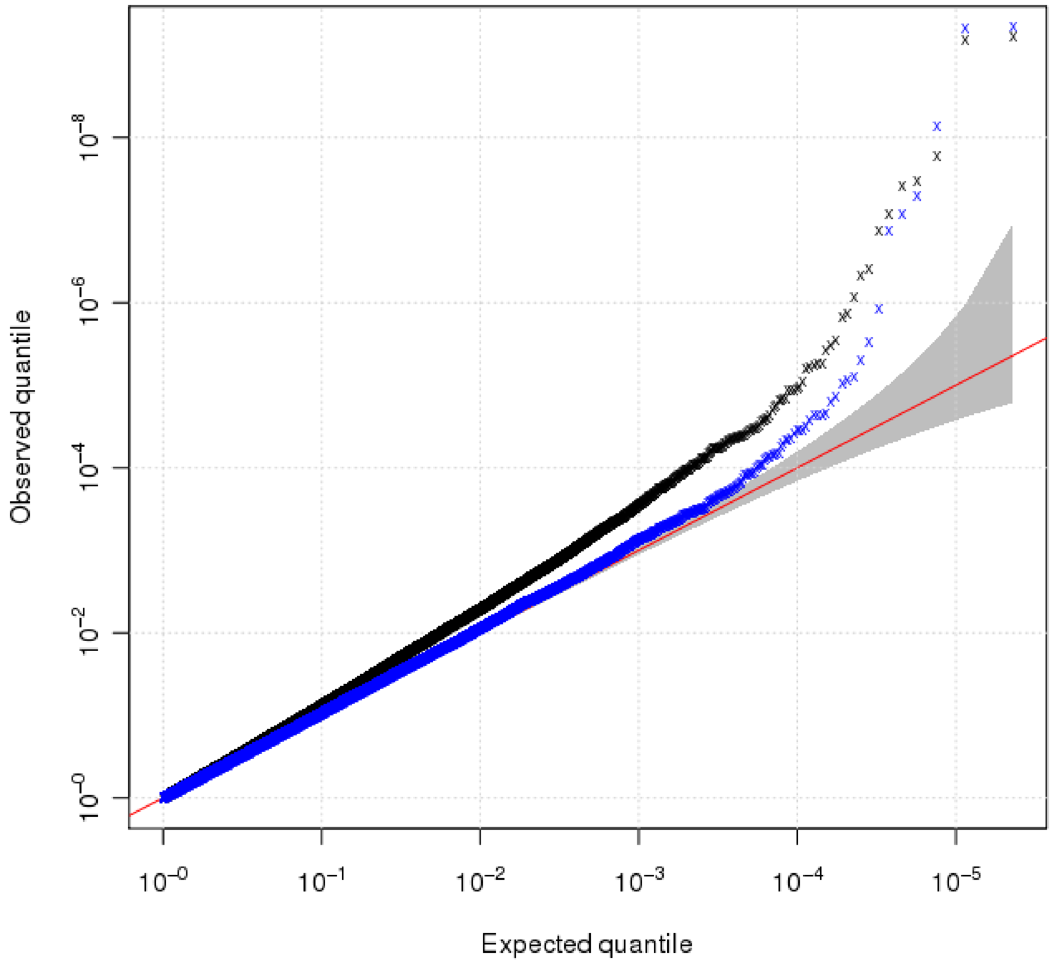

Supplementary Figure S2. Q-Q plots for the joint analysis.
The black shows the unadjusted and the blue the adjusted QQ plot after controlling for population stratification, with the red line showing the expectation under the null hypothesis, and the 95% confidence intervals in the shaded area. Before adjustment, the test statistic is clearly inflated. After adjustment, the test statistic is not inflated: $\lambda_{GC} = 1\cdot04$, and the observed and expected lines tally well. The tail of the adjusted plot deviates upwards, consistent with true positive association.
